# Management of a Patient With Myocardial Infarction With Non-obstructive Coronary Arteries (MINOCA) Secondary to Myopericarditis: A Case Report

**DOI:** 10.7759/cureus.76851

**Published:** 2025-01-03

**Authors:** Namratha Edpuganti, Basil N Nduma, Chukwuyem Ekhator

**Affiliations:** 1 Internal Medicine, Mamata Medical College, Khammam, IND; 2 Internal Medicine, Medical City Hospital, Denton, USA; 3 Neuro-oncology, New York Institute of Technology, College of Osteopathic Medicine, Old Westbury, USA

**Keywords:** acute coronary syndrome (acs), coronary artery embolism, coronary microvascular dysfunction (cmd), coronary vasospasm, ischemia with non-obstructive coronary arteries (inoca), myocardial infarction (mi), myocardial infarction with non-obstructive coronary arteries (minoca), myopericarditis, takotsubo

## Abstract

The diagnosis and management of myocardial infarction with non-obstructive coronary arteries (MINOCA) presents a formidable challenge to clinicians due to its multifaceted etiologies and underlying pathophysiological mechanisms. Etiologies encompass a spectrum including myopericarditis, coronary vasospasm, microvascular diseases, coronary artery embolism, and takotsubo syndrome, among others. Despite its clinical significance, leading medical organizations need more consensus guidelines delineating the optimal approach to MINOCA diagnosis, treatment, and follow-up.

In this case report, we elucidate a complex case of a 65-year-old male devoid of significant cardiovascular history who presented with characteristic chest pain, ST-segment elevation on electrocardiography, and markedly elevated troponin levels. Coronary angiography revealed non-obstructive coronary vessels, posing a diagnostic conundrum. Subsequent literature reviews of advanced imaging modalities such as cardiac magnetic resonance imaging (MRI) and coronary angiography with cardiac biopsy were noted to be pivotal in elucidating the specific etiology of MINOCA, which otherwise posed a diagnostic challenge.

Ultimately, the patient was diagnosed with MINOCA secondary to myopericarditis, underscoring the importance of a comprehensive diagnostic approach in such cases. This case underscores the critical role of advanced imaging techniques in delineating the underlying pathology of MINOCA and emphasizes the necessity for individualized management strategies tailored to the specific etiology. Furthermore, we discuss potential strategies for optimizing the diagnostic workup and discharge planning following coronary angiography in patients with MINOCA.

## Introduction

While most acute coronary syndromes (ACS) are caused by obstructive coronary artery disease (CAD), a subset, constituting 6%-8% of cases, occurs in the context of non-obstructive coronary arteries, referred to as myocardial infarction with non-obstructive coronary arteries (MINOCA) [1]. It denotes an ACS characterized by an angiographically verified absence of obstructed coronary artery or stenosis equal to or less than 50% with no clinically overt specific cause for the acute presentation [1]. The prevalence of MINOCA is approximately 6%-8% of patients experiencing acute myocardial infarction (AMI). However, this reported prevalence exhibits considerable variability, ranging from 3.5% to 15%, which may be attributed to variances in the populations studied and disparities in its definition [2]. Furthermore, MINOCA tends to be more prevalent among younger individuals and women [2]. 

Myocardial infarction with non-obstructive coronary arteries represents a diagnostic challenge in clinical practice, requiring a meticulous approach to exclude other etiologies of ACS. The diagnostic criteria for ACS encompass a constellation of ischemic symptoms, such as typical chest pain, along with biochemical evidence of myocardial injury reflected by elevated cardiac biomarkers, notably troponins. Clinical markers, including electrocardiogram (EKG) and echocardiogram changes, are pivotal in the diagnostic algorithm, comprising new-onset ST/T changes, new left bundle branch block (LBBB), development of pathological Q-waves on EKG, and new loss of viable myocardium or regional wall motion abnormality (RWMA) on imaging [3]. Upon establishing a preliminary assessment of ACS using the criteria above, angiography is pursued to delineate the extent of coronary stenosis. In instances where angiography reveals coronary arteries with <30% stenosis or ≥ 30% but < 50% [4], the patient satisfies the diagnostic criteria for MINOCA. Subsequently, the diagnostic focus shifts toward elucidating the specific etiology underlying MINOCA and implementing targeted treatment strategies both at discharge and throughout the continuum of care, with particular emphasis on cardiac magnetic resonance imaging (MRI) and cardiac biopsy, central themes elucidated in this discourse [2].

## Case presentation

The patient was a 65-year-old male who presented with a one-day history of chest pain, described as sharp and stabbing in nature. The pain was located in the left side of the chest and radiated to the left shoulder. There was no prior episode of similar pain. The pain started while the patient was resting at home and persisted until he was brought by emergency medical services to the emergency room (ER), where he received morphine, which relieved his pain. The patient did not have any atherosclerotic vascular disease risk factors for ACS; he was a lifetime non-smoker, did not have hypertension or diabetes mellitus, was not obese, and denied a family history of cardiovascular or cerebrovascular events. His other medical problems included gastroesophageal reflux disease and a partial hemicolectomy secondary to inflammatory bowel disease. His physical examination findings were grossly unremarkable and documented as follows: The examination of the head, eyes, ears, nose, and throat revealed an atraumatic, normocephalic head. Extraocular muscles were intact, and pupils were equal, round, and reactive to light and accommodation, with normal eye fundi. The nose showed no nasal congestion, and the throat examination revealed no tonsillar erythema, exudates, or enlargement. The mouth had moist mucous membranes, good dentition, and no lesions. The neck was supple with no jugular venous distention, a normal thyroid, and no cervical lymphadenopathy. The nervous system examination showed the patient to be alert and oriented to person, place, and time, with good concentration and cranial nerves II-XII grossly intact. Motor strength was 5/5 in all muscle groups, and sensation was intact to sharp and dull stimuli. Chest and lung examination revealed clear auscultation bilaterally with no rales, rhonchi, wheezing, or rubs, and no tenderness to palpation. The heart examination showed a point of maximal impulse that was not displaced, a regular rate and rhythm, normal S1 and S2 heart sounds, and no murmurs, rubs, or gallops. The abdomen was soft, non-tender, and non-distended, with normal bowel sounds, no hepatosplenomegaly, and an ileostomy bag in situ. Examination of the extremities showed no clubbing, cyanosis, or edema. The mental status exam revealed a normal mood, affect, and judgment. The chest X-ray was unremarkable.

Vital signs were as follows: blood pressure of 142/79 mmHg, heart rate of 73 beats per minute, respiratory rate of 17 breaths per minute, temperature of 37.1°C, and oxygen saturation of 98% on room air. The patient’s weight was 75.7 kg, height 172.72 cm, and body mass index was 25.4 kg/m², which classified him as mildly overweight. Table 1 below shows the laboratory results, including reference values and units of measurement.

**Table 1:** The Patient’s Laboratory Reports mmol/L: millimoles per liter; mg/dL: milligrams per deciliter; U/L: units per liter; pg/mL: picograms per milliliter; g/dL: grams per deciliter; mIU/L: milli-international units per liter; x10^3/µL: thousand per microliter; fL: femtoliters; %: percentage; ng/L: nanograms per liter. Key: (L) - indicates that lab values are low and (H) - indicates that lab values are elevated.

Test	Result	Reference Values	Units
Sodium (Na)	134 (Low)	135-145	mmol/L
Potassium (K)	3.4 (Low)	3.5-5.0	mmol/L
Chloride (Cl)	99	98-106	mmol/L
Bicarbonate (HCO3)	28	22-29	mmol/L
Anion Gap	7	03-Nov	mmol/L
Blood Urea Nitrogen (BUN)	15	7-20	mg/dL
Creatinine (Cr)	1.56 (High)	0.6-1.2	mg/dL
Random Blood Glucose	153	70-99 (fasting)	mg/dL
Corrected Calcium (Ca)	8.9	8.5-10.2	mg/dL
Total Bilirubin (Bil)	0.8	0.1-1.2	mg/dL
Aspartate Aminotransferase (AST)	45 (High)	10-40	U/L
Alanine Aminotransferase (ALT)	44	7-56	U/L
Alkaline Phosphatase (ALP)	55	44-147	U/L
B-type Natriuretic Peptide (BNP)	24.5	<100	pg/mL
Total Protein	7.9	6.3-8.2	g/dL
Albumin	3.8	3.5-5.0	g/dL
Lipase	94	0-160	U/L
Thyroid-Stimulating Hormone (TSH)	1.8	0.4-4.0	mIU/L
White Blood Cell Count (WBC)	6.5	4.0-11.0	x10^3/µL
Hemoglobin (Hgb)	14.4	13.8-17.2	g/dL
Mean Corpuscular Volume (MCV)	86.1	80-100	fL
Platelet Count	125 (Low)	150-450	x10^3/µL
Hemoglobin A1C (HbA1C)	5.9 (Pre-diabetic)	<5.7 (Normal)	%
Triglycerides	158 (High)	<150	mg/dL
Total Cholesterol	173	<200	mg/dL
Low-Density Lipoprotein (LDL)	116	<100	mg/dL
High-Density Lipoprotein (HDL)	25 (Low)	>40	mg/dL
Troponin I (High Sensitivity)	3373 --> 4451 --> 10772	<14	ng/L

**Figure 1:**
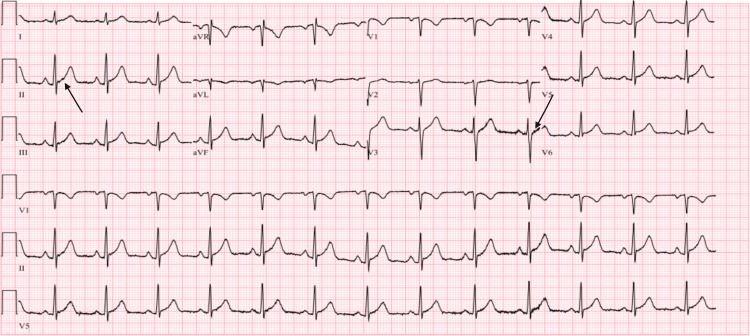
Electrocardiogram (EKG) showed J-point elevation in lead II and, to some extent, in V5 and V6, as indicated by black arrows.

The EKG (Figure 1) showed J-point elevation in lead II and, to some extent, in V5 and V6, as indicated by black arrows. 

The patient was noted to have a thrombolysis in myocardial infarction (TIMI) score of three, based on his age of 65, ST changes on EKG, and elevated troponins, indicating a severe risk for ACS with a 13% risk of all-cause mortality within 14 days. Given these findings, a diagnosis of ACS was made, and the patient was administered a dose of aspirin 325 mg and atorvastatin 40 mg and started on a heparin infusion. A cardiologist was consulted, and the patient was scheduled for coronary angiography with the possibility of stenting. However, the angiography revealed no evidence of aneurysm or significant obstruction in the coronary vessels, with less than 30% stenosis noted.

Assessments post procedure

The patient was diagnosed with non-obstructive CAD, characterized by up to 20% stenosis in the left anterior descending and right coronary artery, alongside mildly elevated left ventricular end-diastolic pressure at 20 mmHg. Despite undergoing coronary angiography, the patient continued to experience constant chest pain, which was managed with acetaminophen, morphine, and Norco (hydrocodone/acetaminophen). A diagnosis of MINOCA, likely secondary to myopericarditis, was made. Consequently, the patient was discharged with a treatment plan that included ibuprofen 600 mg for two weeks and a tapering dose of colchicine for three months, along with a scheduled outpatient cardiac MRI and a follow-up visit with a cardiologist. Six months post discharge, a follow-up call revealed that the patient's chest pain had completely resolved with the prescribed medications, although he had not undergone the cardiac MRI due to expired insurance and financial constraints.

## Discussion

Our primary diagnosis was MINOCA secondary to myopericarditis. The reasons why we concluded on this diagnosis are as follows: persistent and constant atypical chest pain despite coronary angiographic evidence of normal coronary arteries indicating an inflammatory process in the cardiac myocardium and/or pericardium; furthermore, the J-point elevation occurred in multiple cardiac segments, which is consistent with a more diffuse pathologic process like pericarditis or myopericarditis, and finally, the elevated troponins, which is vital in actually diagnosing myopericarditis and differentiating it from acute pericarditis, which does not typically have elevated troponins [5]. In accordance with our thought process, the diagnostic criteria for acute pericarditis include two or more of the following: typical chest pain, detection of a pericardial friction rub during examination, noticeable ECG alterations such as diffuse concave ST-segment elevation coupled with PR depression, or evidence of pericardial effusion [5]. Myopericarditis, on the other hand, is confirmed with the presence of the acute pericarditis features above, along with one of the following additional criteria: elevated levels of cardiac biomarkers, suspected new-onset left ventricular systolic dysfunction as determined by echocardiography or cardiac MRI, or indications of myocardial inflammation via cardiac MRI [5]. 

In the case of the patient discussed in this article, he was discharged from the hospital with nonsteroidal anti-inflammatory drugs (NSAIDs) and colchicine and referred for CMR, which the patient did not do. His myopericarditis was treated blindly. Luckily, the patient’s symptoms resolved before he completed his three-month treatment course with two weeks of ibuprofen 600 mg and three months of tapering doses of colchicine according to the guidelines for treatment of myopericarditis [6], retroactively confirming our diagnosis. One of our emphases in this report is individualized care. If the proper diagnosis is made, the patients will quickly benefit from specific and targeted therapy, which will be more beneficial compared to blanket treatment for ACS, such as atorvastatin, beta-blockers, and anti-platelet medications, which these patients are typically blindly prescribed after they are diagnosed with MINOCA [7-9].

On further research into the care of patients with MINOCA and myopericarditis, we realized the importance of cardiac MRI. A cardiac MRI has emerged as a crucial tool for diagnosing patients with suspected MINOCA. Its timely application aids in determining the underlying cause of myocardial injury, thereby assisting in patient management and risk stratification. With high diagnostic accuracy, a cardiac MRI proves effective in delineating various etiologies in patients presenting with nonspecific symptoms like chest pain and dyspnea, who were subsequently found to have elevated troponin levels. Its multimodal capabilities encompass diverse imaging techniques, facilitating the identification and differentiation of myocardial damage etiologies such as acute myocardial infarction, myocarditis, and stress-induced cardiomyopathy [7,5]. In cases of myopericarditis, a cardiac MRI typically reveals inflammatory changes in the subepicardial or mid-myocardial regions alongside myocardial edema across various vascular territories, contrasting with the subendocardial or transmural myocardial enhancement observed in a single arterial territory in ACS. Additionally, a cardiac MRI facilitates the assessment of left ventricular function [5].

Upon further research specifically into myopericarditis, we found that the gold standard for diagnosis is endomyocardial biopsy. However, its application is restricted due to its poor sensitivity, rendering it uncommon unless patients exhibit inadequate response to medical therapy. Endomyocardial biopsy typically demonstrates a sensitivity ranging from 43% to 64%, accompanied by an overall complication rate of 6%, including a 0.4% incidence of perforation-related mortality [8]. Thus, in the case of our patient, endomyocardial biopsy was completely not an option. This point is highlighted so that clinicians can keep it in mind for patients who continue to deteriorate despite being on optimal guideline therapy. 

Other general investigations that could be provided to patients diagnosed with MINOCA include intracoronary imaging using techniques such as intravascular ultrasound or optical coherence tomography, thrombophilia testing, and provocative testing for coronary vasospasm [2]. These investigations have revealed that approximately 40% of patients diagnosed with MINOCA exhibit signs of plaque disruption. While intravascular ultrasound is effective in highlighting plaque rupture, optical coherence tomography emerges as a superior tool for identifying patients with plaque erosion and may offer enhanced assessment capabilities for individuals with spontaneous coronary artery dissection [2]. Thrombophilia disorders are also detectable in up to 14% of MINOCA patients; however, it is unclear if they were the cause of the MINOCA or just an exacerbating factor [2]. 

## Conclusions

There are currently no clear guidelines to direct physicians in the care of patients with MINOCA, both short- and long-term. The majority of patients diagnosed with MINOCA are on standard treatment for patients with ACS, including statins, beta-blockers, and anti-platelet medications. This case underscores the critical role of an advanced imaging technique (cardiac MRI) in delineating the underlying pathology of MINOCA and emphasizes the necessity for further investigation of patients with MONICA as well as individualized management strategies tailored to the specific etiologies once they are identified. 
